# Editorial: Exploring plant stress responses using non-invasive light techniques

**DOI:** 10.3389/fpls.2026.1826533

**Published:** 2026-03-23

**Authors:** Vladimíra Nožková

**Affiliations:** Department of Chemical Biology, Faculty of Science, Palacký University, Olomouc, Czechia

**Keywords:** interdisciplinarity, light, optical methods, non‑invasive plant monitoring, stress response

Non-invasive optical methods are reshaping how plant scientists observe stress responses: by using light as both probe and signal, researchers can monitor physiological and biochemical dynamics without destructive sampling. This Research Topic gathers six complementary contributions that together illustrate the technical breadth and interdisciplinary value of light-based phenotyping—from autofluorescence time series and hyperspectral indices to biospeckle OCT and targeted light-quality treatments—each translating optical signatures into biologically meaningful insight. This Research Topic collectively demonstrates that, by harnessing light and interdisciplinary expertise, we can non-invasively illuminate plant stress responses and translate optical signals into practical solutions.

The Research Topic opens with the study by Cheng that exemplifies how optical principles intersect with emerging materials science. Although the work focuses on the application of tannic acid–iron nanomaterials to mitigate salt stress in rice, it highlights the central role of reactive oxygen species (ROS) in stress signaling—an area increasingly monitored through optical reporters and imaging techniques. The demonstrated ability of nanomaterials to modulate ROS accumulation aligns with broader efforts to visualize oxidative stress non-invasively, underscoring the potential for integrating nanotechnology with optical diagnostics in plant biology.

A different methodological direction is represented by Das Choudhury et al., who leveraged autofluorescence imaging and machine learning to detect drought stress in *Brassica rapa*. Autofluorescence, arising from endogenous fluorophores such as chlorophylls, phenolics, and cell-wall components, provides a rich multidimensional signal without the need for dyes or external markers. By developing a pixel-level classifier capable of distinguishing stressed from non-stressed tissue, the authors demonstrate how high-throughput phenotyping platforms can extract subtle stress signatures from time-series image sequences. Their work illustrates the power of combining optical sensing with computational analysis to reveal genotype-specific stress responses.

At field scale, Thorp et al. evaluate *in situ* hyperspectral reflectance, spectral vegetation indices (SVIs), and machine learning (ML) for chlorophyll phenotyping in cotton. They emphasize practical trade-offs: while ensemble ML methods achieved high accuracy, SVIs often provided more consistent, transferable estimates across seasons—an important reminder that interpretability and robustness remain central for operational phenotyping.

At the microscopic scale, Tyagi et al. introduced biospeckle optical coherence tomography (bOCT) as a rapid, non-invasive method to assess nanoparticle-induced responses in lentil seeds. bOCT detects dynamic speckle patterns generated by coherent light scattering, providing a window into internal metabolic dynamics. Remarkably, the technique detected physiological responses within 20 hours—far earlier than conventional germination assays. This work demonstrates how biophotonics and plant physiology can intersect to create sensitive diagnostic tools for environmental toxicology, seed biology, and early stress detection.

The contribution by Wu et al. highlights another dimension of light–plant interactions: the regulatory role of far-red light in root regeneration of grafted watermelon seedlings. Although the study focuses on physiological and transcriptomic responses rather than imaging, it reinforces the concept that light is both a probe and a signal. Far-red light modulated oxidative stress, sugar metabolism, and auxin signaling—processes that can be monitored through fluorescence-based reporters and optical assays. This work underscores the multifaceted role of light in shaping plant development and stress adaptation.

Finally, Xi et al. synthesize remote-sensing approaches for orchard nitrogen monitoring, reviewing platforms, spectral indices and inversion algorithms and arguing for multi-source integration and phenology-aware models to improve universality and management relevance. This contribution offers a conceptual framework that complements the imaging-based studies in the Research Topic.

Across these contributions common themes emerge. First, non-invasive optical signals—static spectra, time-series fluorescence, speckle dynamics, and controlled illumination—encode complementary aspects of plant status (pigments, ROS, water relations, internal dynamics, gene-regulated responses). Second, interdisciplinarity is essential: sensor engineering, optics, plant physiology, molecular biology and data science must be combined to convert photons into reliable biological inference. Third, methodological rigor—calibration, feature selection, regularization, and validation across environments and phenologies—determines whether models are transferable and actionable.

Looking forward, the field should prioritize standardized calibration protocols, shared annotated datasets, and hybrid modeling strategies that balance physiological interpretability with machine-learning predictive power. When these elements converge, light-based, non-contact methods will not only accelerate basic discovery but also enable scalable, low-impact monitoring and precision management in agriculture and ecology.

I thank all contributing authors for their rigorous and complementary studies, the reviewers for their constructive comments, and the Editorial Office for their support.

